# Impact of life adversity and gene expression on psychiatric symptoms in children and adolescents: findings from the Brazilian high risk cohort study

**DOI:** 10.3389/fpsyt.2025.1505421

**Published:** 2025-02-13

**Authors:** Vanessa Kiyomi Ota, Adrielle Martins Oliveira, Amanda Victória Gomes Bugiga, Helena B. Conceição, Pedro Alexandre Favoretto Galante, Paula Fontes Asprino, Julia Luiza Schäfer, Mauricio Scopel Hoffmann, Rodrigo Bressan, Elisa Brietzke, Gisele Gus Manfro, Rodrigo Grassi-Oliveira, Ary Gadelha, Luis Augusto Rohde, Euripedes Constantino Miguel, Pedro Mario Pan, Marcos Leite Santoro, Giovanni Abrahao Salum, Carolina Muniz Carvalho, Sintia Iole Belangero

**Affiliations:** ^1^ Laboratory of Integrative Neuroscience (LiNC), Universidade Federal de São Paulo (UNIFESP), São Paulo, Brazil; ^2^ Post-Graduation Program in Psychiatry and Medical Psychology, UNIFESP, São Paulo, Brazil; ^3^ National Institute of Developmental Psychiatry & National Center for Innovation and Research in Mental Health (CISM), Sao Paulo, Brazil; ^4^ Genetics Division, Department of Morphology and Genetics, UNIFESP, São Paulo, Brazil; ^5^ Centro de Oncologia Molecular, Hospital Sírio-Libanês, São Paulo, Brazil; ^6^ National Institute of Developmental Psychiatry & National Center for Innovation and Research in Mental Health (CISM), Porto Alegre, Brazil; ^7^ Department of Psychiatry, Hospital de Clínicas de Porto Alegre, Universidade Federal do Rio Grande do Sul (UFRGS), Porto Alegre, Brazil; ^8^ Department of Neuropsychiatry, Universidade Federal de Santa Maria (UFSM), Santa Maria, Brazil; ^9^ Mental Health Epidemiology Group (MHEG), Universidade Federal de Santa Maria (UFSM), Santa Maria, Brazil; ^10^ Graduate Program in Psychiatry and Behavioral Sciences, UFRGS, Porto Alegre, Brazil; ^11^ Care Policy and Evaluation Centre, London School of Economics and Political Science, London, United Kingdom; ^12^ Department of Psychiatry, Queen’s University School of Medicine, Kingston, ON, Canada; ^13^ Translational Neuropsychiatry Unit, Aarhus University, Aarhus, Denmark; ^14^ ADHD Outpatient Program & Developmental Psychiatry Program, Hospital de Clinicas de Porto Alegre, Federal University of Rio Grande do Sul, Porto Alegre, Brazil; ^15^ Medical Council, Centro Universitário de Jaguariúna (UNIFAJ), Jaguariúna, Brazil; ^16^ Medical Council, Centro Universitário Max Planck (UNIMAX), Indaiatuba, Brazil; ^17^ Departamento de Psiquiatria do Hospital das Clínicas da Faculdade de Medicina da Universidade de São Paulo, São Paulo, Brazil; ^18^ Disciplina de Biologia Molecular, Departamento de Bioquímica, UNIFESP, São Paulo, Brazil; ^19^ Department of Global Initiatives, Child Mind Institute, New York, NY, United States

**Keywords:** psychopathology, transcriptome, internalizing symptoms, externalizing symptoms, genetics, trauma

## Abstract

**Introduction:**

While the influence of both genetic and environmental factors on the development of psychiatric symptoms is well-recognized, the precise nature of their interaction throughout development remains a subject of ongoing debate. This study investigated the association between the expression of 78 candidate genes, previously associated with psychiatric phenotypes, in peripheral blood and both adversity and psychopathology in a sample of 298 young individuals assessed at two time points from the Brazilian High Risk Cohort Study for Mental Conditions (BHRCS).

**Methods:**

Psychopathology was assessed using the Child Behavior Checklist (CBCL), considering the total CBCL, p-factor (i.e. general factor of psychopathology), and internalizing and externalizing symptoms as clinical variables. The life adversities considered in this study includes four composite variables: child maltreatment, stressful life events, threat and deprivation. Gene expression was measured using next-generation sequencing for target genes and differential gene expression was analyzed with the DESeq2 package.

**Results:**

Mixed models revealed six genes associated with internalizing symptoms: NR3C1, HSPBP1, SIN3A, SMAD4, and CRLF3 genes exhibited a negative correlation with these symptoms, while FAR1 gene showed a positive correlation. Additionally, we also found a negative association between USP38 gene expression and externalizing symptoms. Finally, DENND11 and PRRC1 genes were negatively associated with deprivation, a latent factor characterized by neglect, parental absence, and measures of material forms of deprivation. No mediation or moderation effect was observed of gene expression on the association between life adversities and psychiatric symptoms, meaning that they might influence distinct pathways.

**Discussion:**

Among these nine genes, NR3C1, which encodes a glucocorticoid receptor, is by far the most investigated, being associated with depressive symptoms, early life adversity, and stress. While further research is needed to fully understand the complex relationship between gene expression, life adversities, and psychopathology, our findings provide valuable insights into the molecular mechanisms underlying mental disorders.

## 1 Introduction

Psychiatric symptoms frequently co-occur, meaning that having one condition increases the risk of developing another (1). Moreover, psychiatry has struggled to define clear boundaries between disorders, and between normal and pathological variation, suggesting that the diagnostic categories may be inadequate (2).

This has led researchers to increasingly focus on transdiagnostic approaches, such as dimensional models, to better understand the complex presentations of mental health. These models often combine into higher-order factors, like internalizing and externalizing symptoms For instance, the internalizing dimension reflects the overlap between depression and anxiety symptoms, while the externalizing dimension captures disruptive disorders and substance abuse (1). Additionally, a general factor of psychopathology, the “p factor,” has been suggested, which captures the shared variance between these dimensions (1, 3).

Previous research found evidence for both genetic and environmental influences on psychiatric symptoms. Epidemiological studies have shown that almost all forms of psychopathology are partly heritable and polygenic, transcending diagnostic boundaries (2, 4), and supporting a transdiagnostic structure of psychopathology. Also, genome-wide association studies (GWAS) have demonstrated that genetic influences are often pleiotropic, affecting multiple psychiatric outcomes (5, 6). However, recent GWASs on internalizing (7), externalizing symptoms (8), and general psychopathology (9), have shown a low single nucleotide polymorphism (SNP) heritability (1.7% - 23.5%), indicating the influence of other genetic and environmental factors on these dimensions. For instance, data obtained from the Brazilian High-Risk Cohort Study for Mental conditions (BHRCS) demonstrated that an increase in stressful life events (SLE) (10), child maltreatment (CM) (11), or threat and deprivation (12) was associated with elevated psychopathology.

However, the molecular mechanisms underlying the complex interplay of genetic and environmental factors in the development of psychopathology remain a subject of ongoing debate. Alterations in gene expression, as well as epigenetic modifications that regulate it, have emerged as promising candidates to elucidate this intricate relationship, given that: i) genetic variants associated with psychopathology are enriched for regulatory elements, suggesting a role in gene expression modulation (13, 14); and ii) gene expression and epigenetic changes are related to both environmental factors and psychopathology (15–18). Of particular interest, a growing body of research has associated exposure to early-life adversities (ELA) with significant changes in gene expression, particularly in genes involved in inflammation and immune system function (19–21). This compelling evidence points towards altered gene expression in leukocytes as a potential mechanism linking immune dysregulation to the increased risk of poor health outcomes commonly observed in individuals who have experienced ELA (20, 22). Supporting this notion, the BHRCS identified a significant indirect effect of the aggregate expression of four genes related to the hypothalamic-pituitary-adrenal (HPA) axis and inflammation, mediating the association between CM and major depressive disorder (MDD) (16). Beyond the impact of ELA, other environmental stressors, such as food restriction and low socioeconomic status, have also been demonstrated to influence gene expression patterns (23, 24). Additionally, a transcriptome-wide association study revealed significant genotype-by-environment interactions, wherein the effects of psychosocial stressors (i.e., deprivation and cumulative lifetime stress) on the risk of MDD were modulated by imputed gene expression levels in both whole blood and brain tissues (25). However, no study to date has comprehensively investigated the longitudinal dynamics of blood gene expression and its interaction with psychopathology and life adversities, nor the interplay between these factors in a cohort of children and adolescents.

Thus, this study aims to investigate within the BHRCS the longitudinal association between blood candidate gene expression and adversity (i.e. SLE, CM, threat and deprivation) or psychopathology (total Child Behavior Checklist (CBCL) score, p factor, internalizing and externalizing problems). Additionally, we aim to investigate the potential mediation or moderation role of gene expression in the relationship between life adversities and psychiatric symptoms (**Supplementary Figure S8**).

## 2 Methods

### 2.1 Study procedures

**Figure 1:**
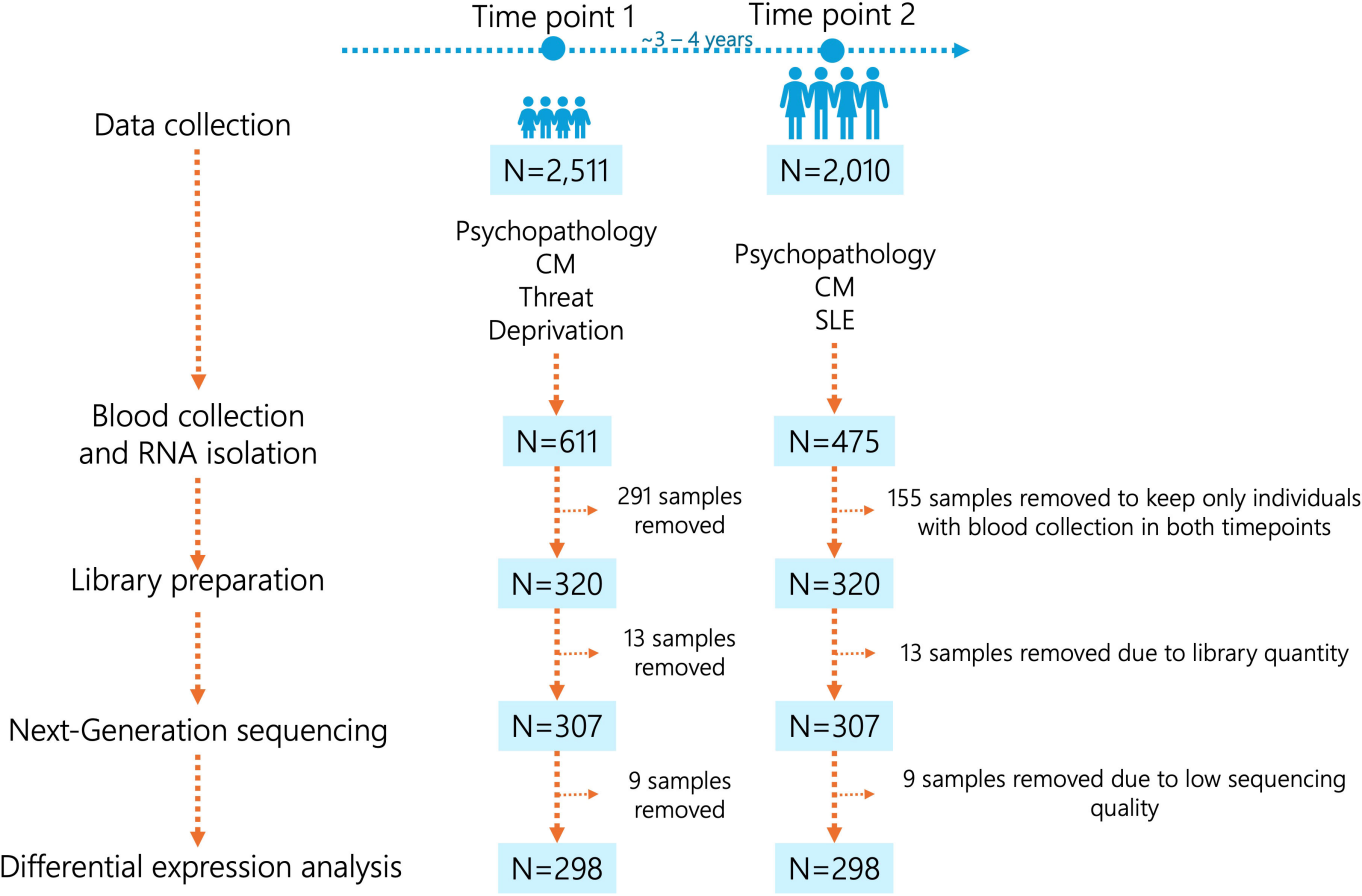
Workflow of the study sample. CM, child maltreatment; SLE, stressful life events.

In this study, we investigated a sample of 320 individuals (**Figure 1**) from the Brazilian High Risk Cohort Study (BHRCS), a large, prospective, community school-based study enriched for high familial risk of psychopathology. Details of the cohort characteristics and study design are described elsewhere (26). Briefly, this cohort is initially comprised 2,511 children and adolescents, with two-thirds presenting a high risk for developing mental conditions based on the presence of early psychiatric symptoms and high family loading of psychopathology. We assessed subjects from two Brazilian cities (Sao Paulo and Porto Alegre) at two different time points: time point 1 (baseline, N=2,511) and time point 2, after 3-year follow-up (N=2,010), with a retention rate of 80%. The total sample comprises children with an average age of 9.7 years, of which 53.1% are male.

For both time points, evaluations were conducted over multiple visits, including a household parent interview and a blood sample collection. The ethics committees of the Institutions involved in the cohort approved the project (the Faculdade de Medicina da Universidade de Saão Paulo (IORG0004884), the Universidade Federal de Saão Paulo (CAAE: 74563817.7.2002.5505), and the Universidade Federal do Rio Grande do Sul (CAAE: 74563817.7.1001.5327). The study was conducted in accordance with approved guidelines and, written informed consent or assent was obtained from all parents and children/youths before their inclusion in the cohort.

### 2.2 Measures of psychopathology

Psychopathology was assessed using the Child Behavior Checklist (CBCL) at all timepoints, including total CBCL score, p-factor, internalizing, and externalizing domains. While the total score and p-factor are highly correlated (the former being the sum of all items, while the latter reflects a non-specific liability to psychopathology), both were examined to determine whether bifactor models better captured biological variation compared to the total score alone. Moreover, the p-factor refers to a shared factor of psychopathology, while the internalizing problems score aggregates anxious-depressed, withdrawn-depressed, and somatic complaints symptoms. The externalizing problems score refers to rule-breaking behavior and aggressive behavior. Confirmatory factor analysis (CFA) was conducted using a bifactor model in which all items are loaded onto a general factor (the p-factor) and residual variance is captured by internalizing and externalizing domains as outlined by the CBCL scoring system (12).

### 2.3 Measures of life adversities

Three environmental variables — stressful life events (SLE), child maltreatment (CM), and threat/deprivation — were constructed based on multiple questionnaires. Detailed information on the generation of these variables is provided in the **Supplementary Information**.

#### 2.3.1 Stressful life events

It was calculated using a Life History Schedule answered by parents at time point 2 (N=2010). This reported exposure to 13 different stressors over the three previous years (between time point 1 and time point 2). Briefly, after exploration of the factor structure using Exploratory Factor Analysis and Confirmatory Factor Analysis (**Supplementary Information**), we used a 1-factor solution comprising nine indicators composed of experiences of family distress (parents’ unemployment, divorce, or death, and family fights, or financial problems), friend or family member’s illnesses or death, loss of a pet and household loss due to a natural disaster (flood or fire).

#### 2.3.2 Child maltreatment

Child maltreatment (CM) was assessed using questionnaires specifically designed for the BHRCS and answered by children and parents covering physical/emotional abuse and neglect, and sexual abuse, as we described in detail previously (11). For the analysis, we used the categorical classification of the children’s level of exposure described in (11).This variable was calculated for all time points (time point 1: N=2511; time point 2: N=2,010).

#### 2.3.3 Threat and deprivation

Threat, defined as the presence of unexpected events endangering the physical integrity or well-being of the child (e.g., abuse, violence), contrasts with deprivation, characterized by neglect, parental absence, and measures of material forms of deprivation (e.g., neglect, poverty). Factor analysis was conducted to assess the latent structure of these experiences at time point 1. Threat was measured using four indicators from The Posttraumatic Stress Disorder (PTSD) assessment of the DAWBA (physical abuse, attack or threat, domestic violence witnessing, attack witnessing); and seven additional questions: Bullying exposure (parent report), Bullying exposure (child report), Physical abuse (parent report), Physical abuse (child report), Emotional abuse (parent report), Emotional abuse (child report), Sexual abuse (total). For deprivation, six indicators were considered to measure deprivation according questionnaires applied in our cohort: Mother’s educational level, socioeconomic classification according to Brazilian Economic Classification Criterion (A/B — the wealthiest, C, or D/E — the poorest), father presence (in contact, noncontact, deceased, or unknown), neglect (parent report), Neglect (child report), Family income. These variables were described in detail previously (12).

### 2.4 Gene expression

We selected only participants with blood samples collected at both time points (N=320). The workflow of the study sample is depicted in **Figure 1**. Blood samples were collected in PAXgene RNA tubes (PreAnalytix, Switzerland), and leukocyte RNA was extracted from whole blood using the PAXgene Blood RNA Kit (Qiagen, Germany). RNA quality and quantity were assessed with the NanoDrop™ 1000 Spectrophotometer (Thermo Fisher Scientific) and the Qubit 2.0 fluorometer with the Qubit RNA BR Assay Kit (Thermo Fisher Scientific), respectively. DNA contamination was verified on a 1.5% agarose gel, and RNA integrity was evaluated using the Agilent 2100 Bioanalyzer and RNA 6000 Nano Kit (Agilent). Gene expression was measured using next-generation sequencing with the TruSeq^®^ Targeted RNA Expression protocol (Illumina). Genes were selected based on four previous studies, two of them in the BHRCS. Briefly, 55 genes exhibited differential expression in the blood of BHRCS individuals who transitioned from low to high psychiatric symptoms over a 3-year follow-up period, indicating the onset of symptoms (15). Conversely, four genes were differentially expressed in individuals who showed a reduction in psychiatric symptoms, suggesting remission (15). Additionally, three genes displayed differential expression in the blood of BHRCS participants with major depressive disorder (16). However, it is important to note that these two previous studies involved a smaller sample size from the BHRCS. Furthermore, we included 20 genes known to be differentially expressed in the brains of individuals with autism spectrum disorders, schizophrenia, bipolar disorder, depression, or general mental health conditions (27), as well as 6 endogenous control genes for blood samples for normalization procedures (28). The probes sequences and references are provided in **Supplementary Table S4**. A total of 100 ng of RNA was reverse transcribed into cDNA using ProtoScript II Reverse Transcriptase (New England Biolabs). Libraries were prepared using the TruSeq Targeted RNA Expression Kit and the TruSeq^®^ Targeted RNA Custom Oligonucleotide Pool (Illumina). Libraries were pooled and sequenced on the NextSeq 500 instrument (Illumina) using the MidOutput v2 kit (150 cycles) and 100 single end reads.

To quantify gene expression, we downloaded the transcript list (manifest file) provided by Illumina (**Supplementary Table S4**). We then converted the target coordinates from the hg19 reference genome to the hg38 reference genome using the liftOver tool. Each target was assigned a unique GENCODE ID by comparing its coordinates with genes annotated by GENCODE [version 38 (29)] using the bedtools intersect tool [default parameters (30)]. To guarantee precise GENCODE ID assignment, a manual verification was performed. Other coding genes that overlapped with the targets in the GENCODE annotated transcripts were removed. Kallisto (31) was used to initiate the quantification of target expression. We generated a transcript index based on this new transcript list (excluding overlapping genes) using the command “kallisto index -i”. Kallisto was then executed for each sample individually (single end mode). The analysis considered the average read length calculated for each sample and a standard deviation of 0.0000001. To quantify gene-level expression, the tximport tool (32) was employed. A pre-filtering step was applied to remove genes with low read counts (less than 10 reads in 10 samples). Read counts were then normalized using the DESeq2 package and the variance stabilizing transformation (VST) function. Finally, outlier values were removed, and the data was standardized.

### 2.5 Statistical analysis

For descriptive analyses comparing time point 1 and time point 2 assessments, we employed paired t-tests for continuous variables and McNemar’s chi-squared tests for categorical variables. Pearson’s correlation tests were conducted to examine the relationships between continuous psychopathological variables and life adversities.

Principal Component Analysis (PCA) was performed to assess potential batch effects in the sequencing data. To investigate the association between gene expression and various factors, we utilized mixed-effect models implemented with the lme() function from the nlme R package. Gene expression, measured using normalized read counts, served as the dependent variable in all analyses. To assess the relationship between gene expression and psychopathology (or CM), we performed separate mixed models for each time point (1 and 2). These models included psychopathology (or CM) as fixed effect, along with sequencing run, age and sex. Data collection site was included as a random effect. To identify longitudinal effects and assess how the relationship between gene expression and psychopathology (or CM) changes over time, we fit mixed-effects models that included data collection site and each subject as random variable. In these models, we included psychopathology (or CM), its interaction with time point, time point, sequencing run, age and sex as fixed effects. This analysis followed the model: gene expression ~ psychopathology (or CM) * time point + sequencing run + age + sex + (1|data collection site/individual ID). For the association between gene expression and SLE, threat, or deprivation, separate analyses were performed for each time point due to differences in data availability: time point 1 for threat and deprivation, and time point 2 for SLE. In these mixed models, the respective stressor was included as the fixed effect, with sequencing run, age, and sex as additional fixed effects, and data collection site as a random effect. To identify potential bias due to changes in gene expression over time, we examined the association between gene expression and time point by including time point, sequencing run, and age as fixed effects, with each subject as a random effect.

Pearson correlation was used to assess correlations between the differentially expressed genes. Network analysis was then performed using GeneMania, considering only co-expression networks, max resultant genes = 5 and max resultant attributes =5, and the automatically selected weighting method (33). To understand the role of the differentially expressed genes, we utilized the gene2func function from the FUMA GWAS software (Functional Mapping and Annotation of Genome-Wide Association Studies) (34) to identify enriched gene sets, such as those in Gene Ontology (GO), Kyoto Encyclopedia of Genes and Genomes (KEGG), and Reactome, as well as gene expression patterns in the Genotype-Tissue Expression (GTEx) version 8 and BrainSpan databases.

We also evaluated the interaction and mediation between gene expression and life adversities on psychopathology using the lavaan package (**Supplementary Figure S8**). The statistical analyses were carried out in R v4.3.3 using RStudio interface. To adjust for multiple comparisons, the Benjamini-Hochberg false discovery rate (FDR) method was applied, using a significance threshold of FDR < 0.05.

## 3 Results

This study included 298 participants from the BHRCS, selected based on high-quality RNA data at both time point 1 and time point 2. The sample comprised more male participants (n=181, 60.7%) and participants from São Paulo state (n=176, 59.1%).

Comparisons between time point 1 and time point 2 are shown in **Table 1**. The prevalence of individuals with high CM significantly decreased from time point 1 (66/22.1%) to time point 2 (43/14.4%; p=0.014). Participant age ranged from 5 to 14 years at time point 1 and 9 to 17 years at time point 2. Internalizing domain scores increased significantly from time point 1 (mean=-0.03, SD=0.30) to time point 2 (mean=0.03, SD=0.32; p<0.001).

**Table 1:** Study population characteristics comparing baseline (time point 1) and follow-up (time point 2).

	Time point 1 (N=298)	Time point 2 (N=298)	p value
Age in years (mean (SD))	9.87 (1.81)	13.14 (1.82)	<0.001
CBCL total score (mean (SD))	26.79 (26.20)	28.41 (24.66)	0.275
p factor factor loading (mean (SD))	0.00 (0.60)	0.06 (0.56)	0.101
Internalizing dimension (mean (SD))	-0.03 (0.30)	0.03 (0.32)	<0.001
Externalizing dimension (mean (SD))	0.02 (0.24)	0.01 (0.24)	0.485
High CM (%)	66 (22.1)	43 (14.4)	0.014
Stressful life events (mean (SD))	NA	0.17 (0.70)	NA
Threat (mean (SD))	0.09 (0.79)	NA	NA
Deprivation (mean (SD))	0.01 (0.80)	NA	NA

CBCL, Child Behavior Checklist; CM, child maltreatment; NA, not applicable.

Additionally, this subsample of 298 participants demonstrated a significant association between life events and psychopathology measures (**Supplementary Figure S2**), consistent with findings from the original study population (12). Also, the threat and deprivation variables calculated for time point 1 are positively correlated with SLE calculated at time point 2 (**Supplementary Figure S3**). Finally, individuals in the high CM group exhibit more SLE, threat and deprivation than those in the low CM group (**Supplementary Figure S4**).

Of the 88 target genes, six endogenous genes and four with low expression (fewer than 10 reads in at least 10 samples) were excluded. This resulted in a final set of 78 genes for differential expression analysis. Principal component analysis revealed an effect of the sequencing run, leading us to include this variable as a covariate in further analysis (**Supplementary Figure S5A**). While no clustering by time point was identified (**Supplementary Figure S5B**), a total of 22 genes exhibited significant associations with time point (**Supplementary Table S5**), suggesting that the expression of these genes might change over time.

We investigated the association between expression levels of the 78 target genes and psychopathology, as measured by the total CBCL score, the p factor, internalizing and externalizing specific factors. Analyzing each time point separately, we observed a significant negative association between *USP38* gene expression and externalizing symptoms at time point 1 (**Table 2**; **Supplementary Figure S6**). At the time point 2, we found significant positive associations between internalizing symptoms and *FAR1* expression; and negative associations between internalizing symptoms and the expression of *NR3C1, HSPBP1, SMAD4, CRLF3* and *SIN3A* genes (**Table 2**; **Supplementary Figure S6**). However, mixed models revealed no longitudinal changes in the relationship between gene expression and psychopathology.

**Table 2:** Significant results from mixed models comparing gene expression and psychopathology or life adversities.

Ensembl ID	Gene	Description	Coefficient	p-value	adjPvalue	Model	Design
ENSG00000170185	*USP38*	Ubiquitin specific peptidase 38	-0.909	5.65E-05	0.004	Mixed model at timepoint 1	~ Externalizing + age + Sex +batch + (1|site)
ENSG00000257093	*DENND11*	DENN Domain Containing 11	-0.173	4.24E-04	0.021	Mixed model at timepoint 1	~ Deprivation + age + Sex +batch + (1|site)
ENSG00000164244	*PRRC1*	Proline Rich Coiled-Coil 1	-0.199	5.36E-04	0.021	Mixed model at timepoint 1	~ Deprivation + age + Sex +batch + (1|site)
ENSG00000113580	*NR3C1*	Nuclear receptor subfamily 3, group C, member 1	-0.581	1.04E-03	0.025	Mixed model at timepoint 2	~ Internalizing + age + Sex +batch + (1|site)
ENSG00000133265	*HSPBP1*	Heat-shock 70-kd protein-binding protein 1	-0.545	1.09E-03	0.025	Mixed model at timepoint 2	~ Internalizing + age + Sex +batch + (1|site)
ENSG00000141646	*SMAD4*	SMAD family member 4	-0.462	1.23E-03	0.025	Mixed model at timepoint 2	~ Internalizing + age + Sex +batch + (1|site)
ENSG00000197601	*FAR1*	Fatty acyl-CoA reductase 1	0.342	1.29E-03	0.025	Mixed model at timepoint 2	~ Internalizing + age + Sex +batch + (1|site)
ENSG00000176390	*CRLF3*	Cytokine Receptor Like Factor 3	-0.507	1.77E-03	0.0285	Mixed model at timepoint 2	~ Internalizing + age + Sex +batch + (1|site)
ENSG00000169375	*SIN3A*	SIN3 transcription regulator family member A	-0.446	2.50E-03	0.03325	Mixed model at timepoint 2	~ Internalizing + age + Sex +batch + (1|site)

Regarding the association between gene expression and measures of life adversities, we identified a negative correlation at time point 1 between deprivation and the expression of both *DENND11* (or *KIAA1147*) and *PRRC1* (**Table 2**; **Supplementary Figure S6**). No associations with other measures of life adversities were identified.

Notably, all nine genes were correlated with each other (**Supplementary Figure S7A**), suggesting potential co-expression or involvement in shared pathways. GeneMania analysis confirmed this, showing that all genes except *HSPBP1* are related by co-expression networks (**Supplementary Figure S7B**). In particular, *NR3C1, SIN3A*, and *SMAD4*, all associated with internalizing symptoms, are correlated with each other (*NR3C1-SIN3A*: r=0.594, p=2.2E-16; *NR3C1-SMAD4*: r=0.673, p=2.2E-16; *SIN3A-SMAD4*: r=0.797, p=2.2E-16; **Supplementary Figure S7A**). Furthermore, these three genes are enriched in two Reactome pathways: FOXO-mediated transcription of oxidative stress, metabolic, and neuronal genes (R-HSA-9615017; 3/29 genes, p=1.277E-07, FDR=3.946E-04) and FOXO-mediated transcription (R-HSA-9614085; 3/64 genes; p=1.447E-06, FDR=0.001).

Analysis of gene expression databases (**Supplementary Figures S7C, D**) revealed ubiquitous expression of *HSPBP1* (average log2TPM from 3.527 in blood to 5.628 in the uterus; average log2TPM of 5.056 across multiple tissues), with high levels also observed in brain tissues (average log2TPM=5.345). Moreover, *DENND11* (average log2TPM = 4.058), *PRRC1* (average log2TPM = 3.740), *SIN3A* (average log2TPM = 3.870), *NR3C1* (average log2TPM = 4.002), *SMAD4* (average log2TPM = 4.332), and *FAR1* (average log2TPM = 4.536) were also highly expressed in multiple tissues. Of note, the average expression across multiple tissues was particularly similar for *NR3C1, SMAD4*, and *SIN3A* genes (**Supplementary Figure S7C**). Interestingly, *USP38* expression appears to be more tissue-specific, being highly expressed in muscle (average log2TPM=4.297), whereas *CRLF3* is lowly expressed in this tissue (average log2TPM=1.398) and highly expressed in the spleen (average log2TPM=4.406).

Analysis of gene expression in the BrainSpan database (**Supplementary Figure S7D**) showed that *HSPBP1* is also highly expressed in the brain across all developmental stages (average log2RPKM =5.146 in multiple developmental stages), similar to *DENND11*, although the latter showed more moderate expression (average log2RPKM =3.331 in multiple developmental stages). Notably, while the expression of *SIN3A, SMAD4*, and *PRRC1* appeared higher in prenatal stages (peaking in early prenatal stages: *SIN3A* log2RPKM=3.878, *SMAD4* log2RPKM=4.232, *PRRC1* log2RPKM=3.336), *NR3C1* expression increased in postnatal stages (peaking in young adulthood: log2RPKM=3.222), similar to *FAR1* (peaking in young adulthood, log2RPKM=3.191).

We investigated whether the nine genes were mediators or moderators between the association of life adversities and psychiatric symptoms (**Supplementary Figure S8**). We tested four mediation or moderation models (considering CM, SLE, threat, and deprivation) for each of the *NR3C1, SIN3A, SMAD4, FAR1, CRLF3*, *HSPBP1* and *USP38* genes, which were associated with psychiatric symptoms. We also tested four mediation or moderation models (considering total CBCL score, p-factor, internalizing, and externalizing symptoms) for each of the *DENND11* and *PRRC1* genes, which were associated with deprivation. This resulted in a total of 36 mediation and 36 moderation models tested. We identified *HSPBP1* as a mediator of the association between CM and internalizing symptoms at time point 2 (estimate =0.013; p=0.048). Moreover, we found that *DENND11* expression moderated the relationship between deprivation and the p factor (estimate = -0.080, p = 0.0407), *CRLF3* expression moderated the relationship between CM and internalizing symptoms (estimate = -0.044, p = 0.043), and *NR3C1* expression moderated the relationship between deprivation and internalizing symptoms (estimate = -0.049, p = 0.029) at time point 2. However, these associations were not significant after adjusting for multiple comparisons (adjusted p = 1.000).

## 4 Discussion

We conducted targeted RNA sequencing to identify genes associated with changes in psychopathology and life adversities. Of the 78 genes examined, six genes (*NR3C1, SMAD4, SIN3A, FAR1, CRLF3* and *HSPBP1*) were associated with internalizing symptoms and one (*USP38*) with externalizing symptoms. Additionally, we identified two genes (*DENND11* and *PRRC1*) associated with deprivation, a latent factor constructed from variables such as socioeconomic status, family income, mother’s educational level, neglect, and father presence. Seven genes (*SMAD4, SIN3A, CRLF3, USP38, FAR1, DENND11* and *PRRC1*) were selected based on our previous microarray analysis in the Sao Paulo subsample of the BHRCS (N=103 at baseline and N=103 at follow-up). The results from the current study align with our previous findings, with *SMAD4, SIN3A, CRLF3, USP38, DENND11* (previously known as *KIAA1147*) and *PRRC1* expression decreasing in individuals who transitioned from low to high psychopathology (as measured by the CBCL) (**Supplementary Table S2** from (15)). Consistent with this, the first three were negatively associated with internalizing symptoms, *USP38* with externalizing symptoms and *DENND11* and *PRRC1* were negatively correlated with deprivation in the present study (**Table 2**). *FAR1*, on the other hand, showed increased expression in individuals who transitioned from high to low psychopathology (**Supplementary Table S3** from (15)), contrasting with the positive correlation between *FAR1* expression and internalizing symptoms observed at time point 2 in the current study. Finally, both *NR3C1* and *HSPBP1* genes were selected because they were differentially expressed in the brains of individuals with mental disorders (27), with *NR3C1* also being differentially expressed in the blood of BHRCS individuals with major depressive disorder (16). After adjusting for multiple comparisons, no significant mediation or moderation effects of gene expression on the association between life adversities and psychiatric symptoms were found for these genes.

Adolescence is a period that is particularly vulnerable to environmental influences, and traumatic experiences that occur before or during this stage can significantly impact the likelihood of developing externalizing or internalizing behaviors. Such experiences leave a lasting impact on various biological systems, affecting neuronal, endocrine, immune, transcriptomic, and epigenetic processes (35). Emerging research in genomics, epigenomics, and transcriptomics aims to understand why some individuals are more susceptible to developing certain mental health issues after experiencing trauma (36, 37). For instance, a transcriptome-wide study found that the expression of genes related to the immune system may mediate the link between emotional abuse and MDD (38). Additionally, studies in animal models have shown that patterns of gene expression in cortical and amygdala pyramidal neurons can predict whether fear extinction will be adaptive or maladaptive. which can help to understand trauma-related disorders (39). These findings highlight the complex interactions between biological and environmental factors in shaping mental health outcomes during adolescence.

Notably, traumatic events can disrupt the activity of the HPA axis, which is reflected in cortisol levels. This disruption can lead to changes in functional connectivity in the brain and is associated with internalizing problems later in life (40). Cortisol activates glucocorticoid receptors, which are expressed throughout the brain and can function as transcription factors, regulating genes related to metabolism and immune function (41). In our study, we found the *NR3C1* gene (nuclear receptor subfamily 3, group C, member 1), which encodes the glucocorticoid receptor, to be associated with internalizing. Many studies have associated it with CM and depressive symptoms, particularly those investigating *NR3C1* methylation (42, 43). One of the first studies to investigate this in humans observed decreased expression of *NR3C1*, along with increased methylation of its promoter, in the hippocampus of suicide victims with a history of childhood abuse (44). Changes in *NR3C1* gene expression in blood have also been detected, with some studies identifying increased expression in patients with depression (45) and others finding a negative correlation with depressive scores (46). Our previous study in the BHRCS at time point 1 evaluated gene expression in 20 children and adolescents with major depressive disorder (MDD), 49 with no MDD but with high levels of depressive symptoms, and 61 healthy controls. This study identified decreased expression of *NR3C1* in those with MDD compared to both the depressive symptom group and healthy controls (16), corroborating our current findings at time point 2.

The *SIN3A* gene (SIN3 transcription regulator family member A) encodes a transcriptional regulatory protein. It appears to play an important role in cell cycle events and the proliferation of embryonic stem cells, and it is highly expressed throughout brain development (47), consistent with our analysis in BrainSpan. Indeed, functional studies have revealed that *SIN3A* is a key regulator of cortical expansion and maturation, and its haploinsufficiency has been associated with intellectual disability and autism spectrum disorder (47). Apart from increased SIN3A protein levels in the hippocampus of suicide victims (48), no other association of this gene with mental disorders has been reported, particularly in blood.

The *SMAD4* (SMAD family member 4) gene encodes a member of the SMAD family of signal transduction proteins. It is involved in signal transduction of the transforming growth factor beta (TGFβ) superfamily and pathogenic variants in this gene cause Myhre syndrome, which can include neurobehavioral phenotypes such as developmental delay, autism spectrum disorder, attention-deficit/hyperactivity disorder, and anxiety (49). Moreover, these mutations also disrupt neuronal morphogenesis in both mouse and human neurons (50). The TGFβ pathway has been associated with depression and stress, as it is an anti-inflammatory signal that exerts neuroprotective effects and influences memory formation and synaptic plasticity (51). Preliminary analysis showed that children with lower *SMAD4* expression (predicted by genetic variants) exhibited a positive correlation between prenatal maternal depressive symptoms and amygdala volumes, while those with higher *SMAD4* expression presented a negative correlation between prenatal maternal depression and amygdala volumes (52).

The *FAR1* gene (fatty acyl-CoA reductase 1) encodes a rate-limiting enzyme in plasmalogen biosynthesis (53). Mutations in this gene have been identified in individuals affected by severe intellectual disability, early-onset epilepsy, microcephaly, congenital cataracts, growth retardation, and spasticity (54, 55). Although little is known about this gene, plasmalogens are abundant in the myelin sheath, and thus, plasmalogen homeostasis is likely important for myelination in different regions of the central nervous system (53). Indeed, impaired plasmalogen synthesis has already been implicated in schizophrenia (56–58). Interestingly, the role of *FAR1* in psychopathology appears complex. In a previous study, we found that *FAR1* expression was increased in individuals who transitioned from high to low psychopathology, as measured by the total CBCL score (15). However, in the current study, *FAR1* expression was positively correlated with internalizing symptoms, suggesting a potential association with increased internalizing problems. This apparent discrepancy may be due to several factors, including differences in sample size and the distinct biological underpinnings of internalizing symptoms compared to the broader measure of psychopathology used previously. It highlights the need for further research to fully elucidate the role of *FAR1* in different facets of psychopathology and across different developmental stages.

The *CRLF3* gene (cytokine receptor-like factor 3) encodes a cytokine receptor-like factor. Studies in human and mouse cells have revealed that it is a critical regulator of neurogenesis, neuron survival, and dendritic development, and its deletion appears to cause developmental delay, intellectual disability, and autistic traits (59, 60). Although few studies have investigated this gene, its expression appears to be associated with Alzheimer’s disease (61) and epigenetic age acceleration (62).

The *HSPBP1* gene (heat-shock 70-kd protein-binding protein 1) encodes a cochaperone of heat shock protein 70 (Hsp70) that inhibits the activity of Hsp70 ATPase. It is highly expressed in neurons but lowly expressed in astrocytes. Knocking down HspBP1 in neurons rescued neuropathology in a Huntington’s disease mouse model (63). Although no study investigated this particular gene in relation to mental disorders, alterations in Hsp70 have been reported in bipolar disorder (64) and MDD (65), and medications such as lithium and clozapine appear to alter Hsp70 levels (65).

The *USP38* (Ubiquitin Specific Peptidase 38) gene encodes a deubiquitinating enzyme that acts on different cellular processes, such as the DNA repair, regulation of the cell cycle, and the immune response (66, 67). Recently, the variant rs7681616-C present in the *USP38* gene was associated with an increase in the risk of developing schizophrenia (68), suggesting the importance of this region in psychopathology. Another study identified that *USP38* blood expression was significantly downregulated in schizophrenia patients compared to biological siblings and unaffected controls (69), aligning with our findings of decreased *USP38* expression in individuals with increased externalizing symptoms. Furthermore, a CpG site (cg01769344) located in a CpG island near the *USP38* gene was identified within a co-methylated module associated with general psychopathology in a sample of 440 children from the Generation R cohort (18). Based on this evidence, we suggest that the *USP38* gene may play a significant role in psychopathology.

While our knowledge of the *DENND11* gene (formerly *KIAA1147*) remains limited, its protein product is predicted to participate in regulation of catalytic activity. By similarity, *DENND11* is hypothesized to play a role in neuritogenesis (70), aligning with its elevated expression in multiple brain developmental stages and tissues (**Supplementary Figures S7 C, D**). Furthermore, abnormal DNA methylation levels and the presence of risk-associated single nucleotide polymorphisms (SNPs) have led to its consideration as a candidate gene for frontotemporal dementia with amyotrophic lateral sclerosis (71). Even less is known about the *PRRC1* gene. It is predicted to be involved in the activation of protein kinase A activity and was previously associated with fluid intelligence in a genome-wide association study (GWAS) (72). Interestingly, the same study found that methylation of this gene in the temporal cortex was also associated with fluid intelligence.

We observed correlations among these nine genes (**Supplementary Figure S7A**); however, *HSPBP1* did not appear to be correlated within the co-expression networks (**Supplementary Figure S7B**), which may be explained by its lower expression in blood (**Supplementary Figure S6B**, **S7C**). Of note, *SIN3A, SMAD4*, and *NR3C1*, despite being highly correlated (**Supplementary Figure S7A**), seem to exhibit similar expression patterns in multiple tissues (**Supplementary Figure S7C**) and are involved in two Reactome pathways: FOXO-mediated transcription of oxidative stress, metabolic, and neuronal genes, and FOXO-mediated transcription. None of the genes were associated with time point (**Supplementary Table S5**) or with psychopathology in longitudinal models. This suggests that these results might be time-specific, particularly for internalizing symptoms, considering their increase at time point 2, when the mean age was 13 years (**Table 1**).

The results of this study should be interpreted in light of certain limitations. First, the relatively small sample size may be an issue, warranting further replication studies. While few studies have investigated gene expression in longitudinal children and adolescent cohorts (73), most existing studies in this type of cohort (such as Generation R and Avon Longitudinal Study of Parents and Children) have focused on DNA variations, such as in GWAS (7–9), or DNA methylation (18). Second, this targeted RNA sequencing approach may have missed other genes relevant to psychopathology or be biased by cell type composition. Comprehensive transcriptome analysis could provide a more complete picture in future studies. Third, gene expression is tissue-specific, and our findings do not accurately reflect gene expression changes in the brain. Therefore, these results are more likely to be potential biomarkers of trait or state, and may not be clues about the pathophysiology. Furthermore, longitudinal gene expression analysis is not feasible in brain tissues. Finally, it is uncertain whether other factors that may influence gene expression, such as puberty or infection may have, affected our results, despite adjusting our analyses for sex, age, and time point.

In conclusion, our study has shed light on the complex interplay between genes, life adversities, and psychopathology in young individuals. We identified six genes associated with internalizing symptoms: *NR3C1, HSPBP1, SIN3A, SMAD4*, and *CRLF3* exhibited a negative correlation with these symptoms, while *FAR1* showed a positive correlation. Notably, *NR3C1* has been consistently associated with internalizing symptoms and traumatic events, corroborating our findings. Additionally, decreased *USP38* expression was associated with more externalizing problems, suggesting its possible role in regulating behavioral control or emotional reactivity. Furthermore, we observed lower expression of *DENND11* and *PRRC1* correlated with higher levels of deprivation, indicating a potential role in the biological response to environmental adversity. While the exact mechanisms remain unclear, these findings underscore the complex interplay between genetic predisposition and environmental stressors in shaping mental health outcomes. Although life adversities undeniably contribute to the development of psychiatric symptoms, our study did not find evidence for a direct mediating or moderating effect of gene expression on this relationship. This suggests that the influence of these genes on psychopathology may operate through independent pathways or interact with environmental factors in more nuanced ways. Importantly, our findings highlight the potential of specific gene expression patterns as early indicators of psychiatric vulnerability. The associations observed for *NR3C1, HSPBP1, SIN3A, SMAD4, CRLF3*, *FAR1* and USP38 with internalizing and externalizing symptoms across multiple time points suggest their potential utility in predicting future mental health trajectories. Similarly, the association between *DENND11* and *PRRC1* expression and deprivation underscores the importance of considering both genetic and environmental factors in understanding and addressing mental health challenges.

## Data Availability

RNA sequencing data were deposited into the Gene Expression Omnibus database under accession number GSE288870.
