# Supplementary material for: Impact of life adversity and gene expression on psychiatric symptoms in children and adolescents: findings from the Brazilian high risk cohort study
Source: Front Psychiatry. 2025 Feb 13;16:1505421. doi: 10.3389/fpsyt.2025.1505421 (PMC11866055; doi:10.3389/fpsyt.2025.1505421)
Supplement: Supplementary file 1 [file DataSheet1.docx]

1. **Supplementary Information**

Measures of life adversities:

Three environmental variables were generated based on multiple questionnaires: stressful life events (SLE), child maltreatment (CM) and threat/deprivation. Except for SLE, all the other variables were previously described elsewhere (1,2).

1. ***Stressful life events (SLE)***

The Stressful Life Events (SLEs) variable was calculated using a Life History Schedule answered by the children’s parents at wave 1 (W1, or 3-year follow-up) (N=2010). Parents reported on the exposure to different SLEs Supplementary Table S1 over the three years prior to evaluation, which reflects the time interval between the BHRCS waves.

Exploratory factor analyses (EFA) were conducted using the sample from the state of Rio Grande do Sul as a discovery sample extracting four, three, two, and one factor. The one-factor solution was chosen excluding items that did not load significantly into this factor, yielding a nine-indicator model. A confirmatory factor analysis (CFA) was first conducted with the sample from the state of São Paulo at the W1 (CFI = 0.979, TLI = 0.970, RMSEA = 0.017) and then with the whole sample at the W1 (CFI = 0.953, TLI = 0.932, RMSEA = 0.026). Factor loadings are presented in the Supplementary Table S2 and the final model is depicted in the Supplementary Figure S1.

1. ***Child maltreatment (CM)***

Child maltreatment (CM) was assessed using questionnaires specifically designed for the BHRCS and completed by both children and their parents. These questionnaires targeted four categories of CM recognized in the literature: a) physical abuse, b) neglect, c) emotional maltreatment, and d) sexual abuse.

Trained lay interviewers posed the following questions to parents:

- Has [child's name] ever been seriously beaten by an adult (including yourself) at home, causing injury or leaving bruises or marks?
- Has [child's name] ever not had enough to eat or been forced to wear dirty or torn clothes?
- Has [child's name] ever been called names like "stupid," "idiot," "dumb," or "useless," or been subjected to someone shouting or screaming at them?
- Has anyone ever engaged in sexual activity with [child's name] or threatened to harm them if they refused to do so?

The first three questions were rephrased for the children and administered by trained clinical psychologists:

- Have you ever been seriously beaten by an adult at home, causing injury or leaving bruises or marks?
- Have you ever not had enough to eat or been forced to wear dirty or torn clothes?
- Have you ever been called names like "stupid," "idiot," "dumb," or "useless," or been subjected to someone shouting or screaming at you?

Responses to these seven questions were scored on a 4-point scale: 0 (never), 1 (once or twice), 2 (sometimes), 3 (frequently).

For our analysis, we adopted the categorical classification of children's exposure levels described in (1), calculated for all waves (W0: N=2512; W1: N=2,010). Individuals were categorized into the high exposure trauma group if they reported high exposure to any type of CM. In parents' reports, high exposure was defined as: physical abuse and physical neglect rated "sometimes" or "frequently," sexual abuse rated "rarely," "sometimes," or "frequently," and emotional abuse rated "frequently”.  In children's reports, high exposure was defined as: physical abuse rated "sometimes" or "frequently," physical neglect rated "rarely," "sometimes," or "frequently," and emotional abuse rated "frequently".

1. ***Threat/deprivation***

Threat, defined as the presence of unexpected events endangering the physical integrity or well-being of the child (e.g., abuse, violence), contrasts with deprivation, characterized by the absence of expected social, cognitive, and emotional inputs crucial for development (e.g.,neglect, poverty). Factor analysis was conducted to assess the latent structure of these experiences at baseline (W0) for all subjects (N=2,511). Threat was measured using four indicators from The Posttraumatic Stress Disorder (PTSD) assessment of the DAWBA (physical abuse, attack or threat, domestic violence witnessing, attack witnessing); and seven additional questions (Bullying exposure (parent report), Bullying exposure (child report), Physical abuse (parent report), Physical abuse (child report), Emotional abuse (parent report), Emotional abuse (child report), Sexual abuse (total)). For deprivation, six indicators were considered to measure deprivation according questionnaires applied in our cohort: Mother's educational level, socioeconomic classification according to Brazilian Economic Classification Criterion (A/B—the wealthiest, C, or D/E—the poorest), father presence (in contact, noncontact, deceased, or unknown), Neglect (parent report), Neglect (child report), Family income (2). The factor loading of these latent variables are provided in Supplementary Table S3.

1. **Supplementary Figures**


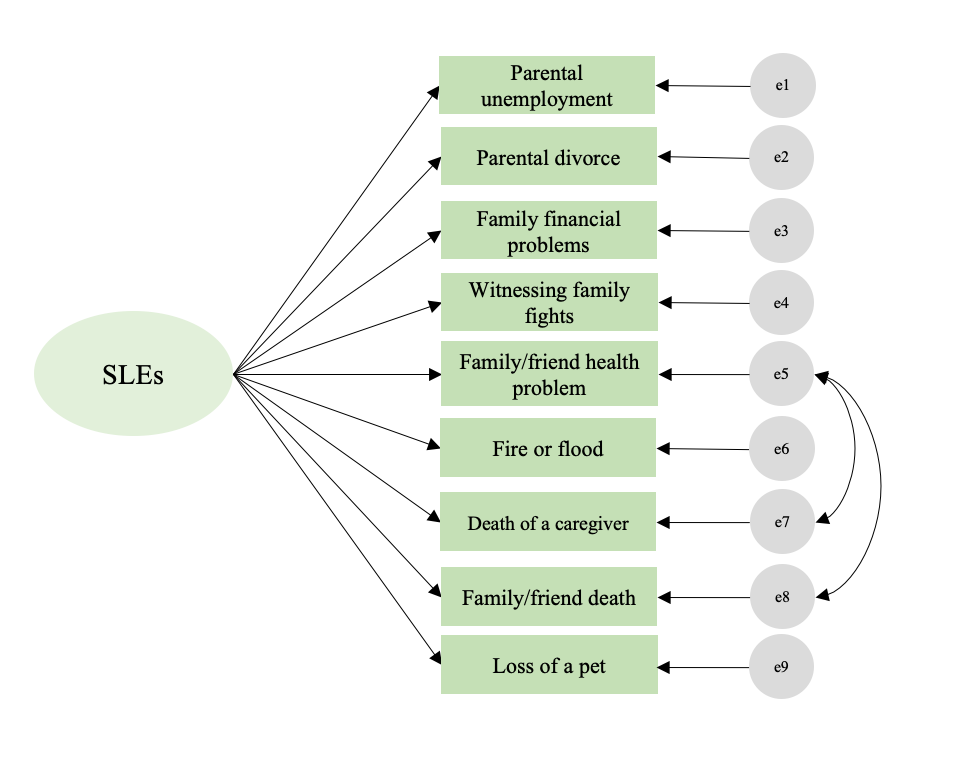


**Supplementary Figure S1:** Stressful Life Events (SLEs) model depiction


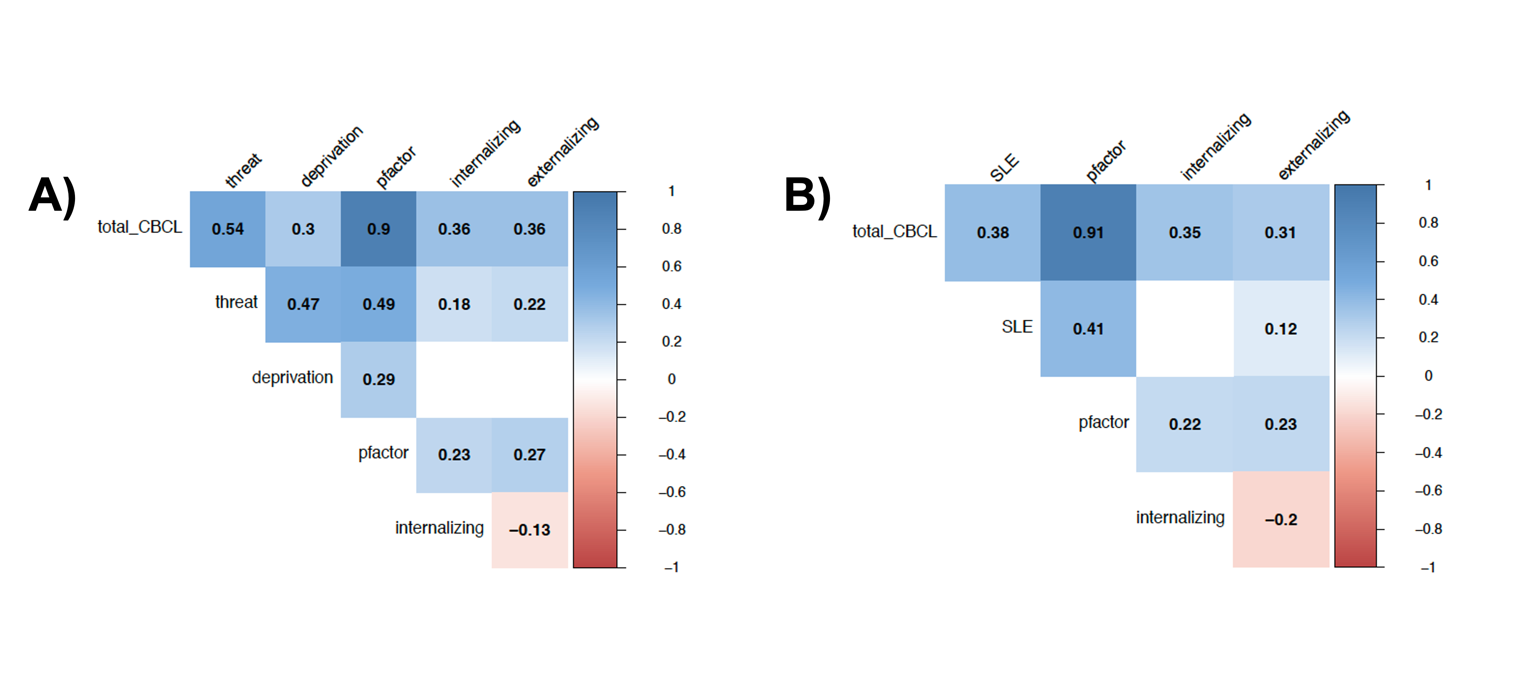


**Supplementary Figure S2:** Correlation between life adversity measures (threat, deprivation, and stressful life events (SLE)) and psychopathology (total CBCL, p factor, internalizing and externalizing domains) at timepoint 1 (A) and timepoint 2 (B). Blue boxes indicate significant positive correlations (Pearson’s r); red boxes indicate significant negative correlations (Pearson’s r); blank boxes indicate non-significant correlations (p>0.05).


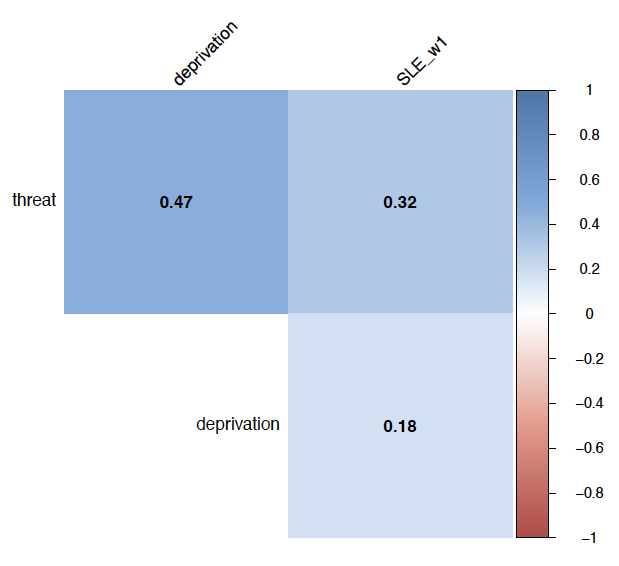


**Supplementary Figure S3:** Correlation between life adversity measures (threat, deprivation, and stressful life events (SLE)) considering threat and deprivation measured at timepoint 1 and SLE measured at timepoint 2 (wave 1 – w1). Blue boxes indicate significant positive correlations (Pearson’s r).

**Supplementary Figure S4:** Boxplots showing that individuals with high child maltreatment also exhibit more stressful life events (stress) at timepoint 2 (left), threat (middle) and deprivation (depriv) (right) at timepoint 1.


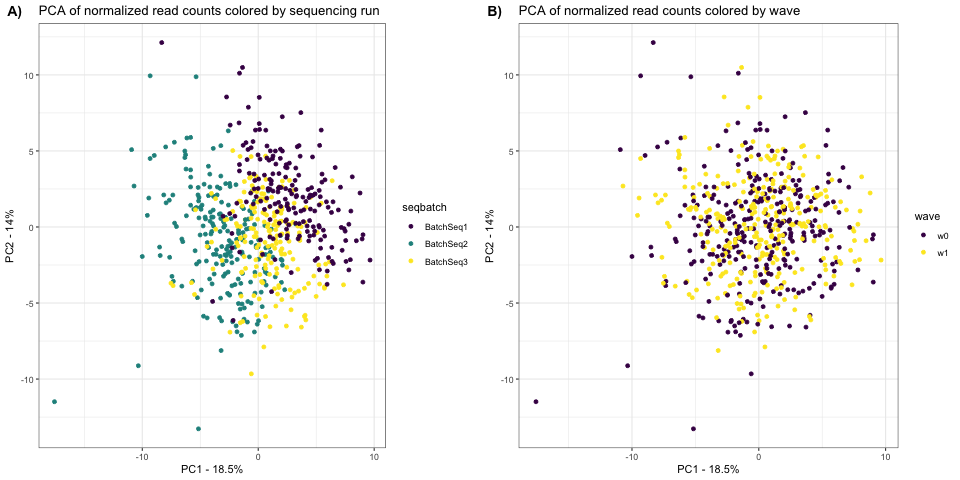


**Supplementary Figure S5:** Principal Component Analysis (PCA) of gene expression colored by sequencing run (A) or timepoint/wave (B).

**Supplementary Figure S6:** Scatter plots illustrating the relationship between internalizing symptoms at time point 2 and the expression levels of *NR3C1* (A), *HSPBP1* (B), *SMAD4* (C), *FAR1* (D), *CRLF3* (E), and *SIN3A* (F). Correlations between externalizing symptoms at time point 1 and *USP38* expression (G), and between deprivation at time point 1 and *DENND11* (H) and *PRRC1* (I) expression are also shown.


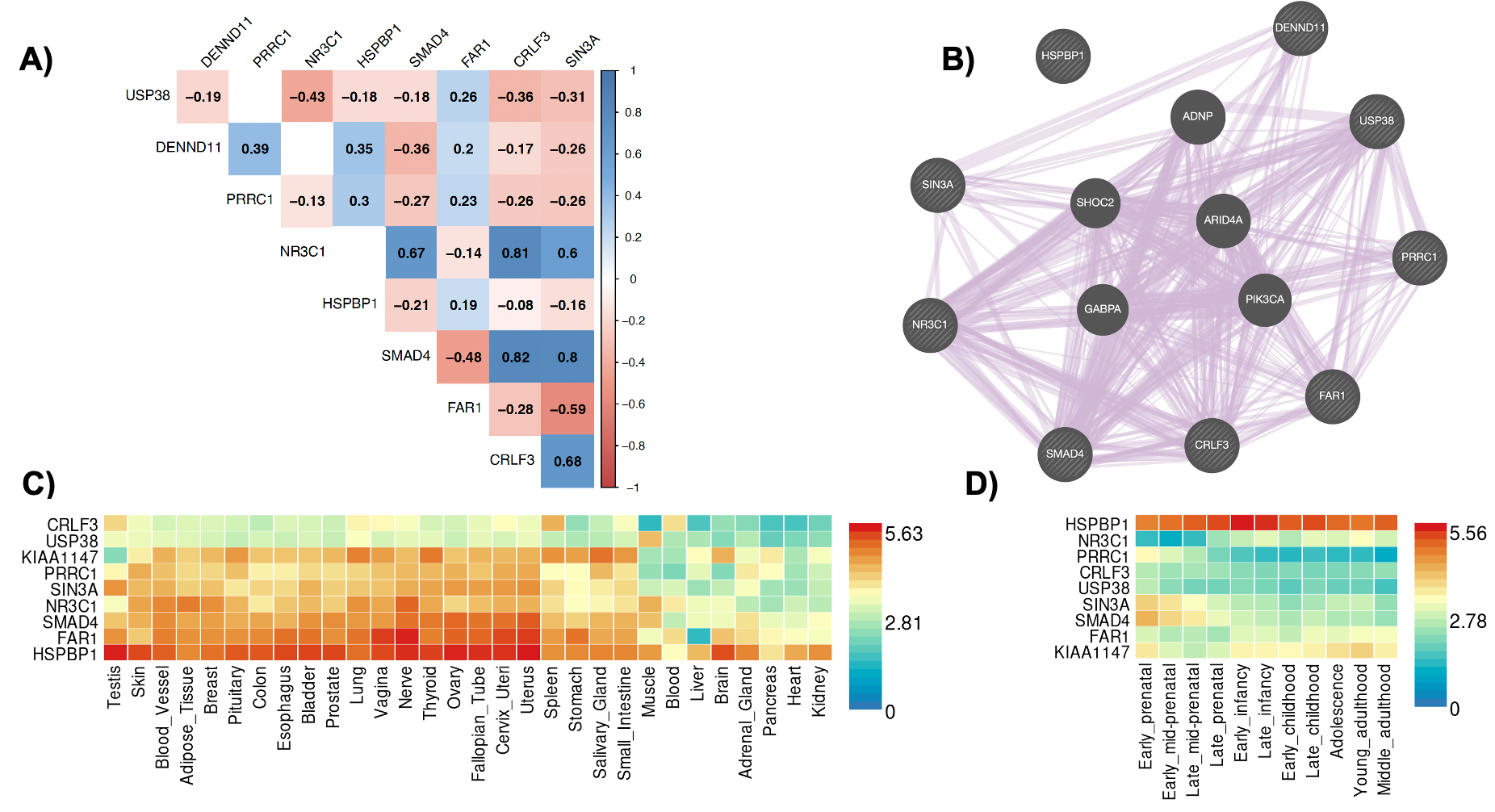


**Supplementary Figure S7: A)** Correlation between the nine differentially expressed genes considering both waves. Blue boxes indicate significant positive correlations (Pearson’s r); red boxes indicate significant negative correlations (Pearson’s r); blank boxes indicate non-significant correlations (p>0.05). **B)** GeneMania network analysis of *NR3C1, SIN3A, SMAD4, FAR1, CRLF3, HSPBP1, USP38, DENND11*, and *PRRC1* (hatched). Purple lines indicate co-expression interactions. **C)** Gene expression heatmaps from the FUMA GWAS software (Functional Mapping and Annotation of Genome-Wide Association Studies) considering the Genotype-Tissue Expression (GTEx) v8 database. Expression values are average expression per label (log2TPM). **D)** Gene expression heatmaps from the FUMA GWAS software considering the BrainSpan database. Expression values are average expression per label (log2 RPKM).


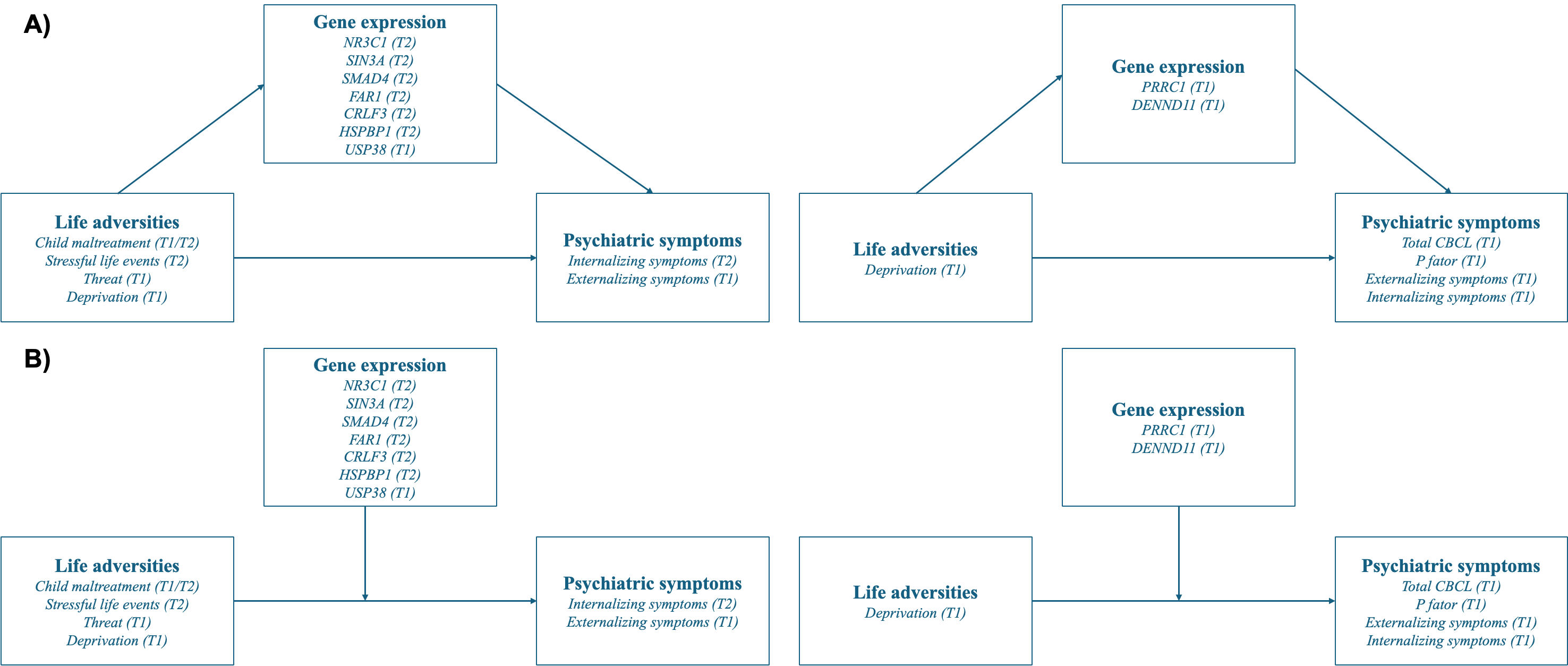


**Supplementary Figure S8:** (A) Mediation and (B) moderation models tested in this study. Variables were assessed at time point 1 (T1) and time point 2 (T2).

**References for Supplementary Information**

1. Salum GA, DeSousa DA, Manfro GG, Pan PM, Gadelha A, Brietzke E, et al. Measuring child maltreatment using multi-informant survey data: a higher-order confirmatory factor analysis. Trends Psychiatry Psychother. 2016;38(1):23–32.

2. Schäfer JL, McLaughlin KA, Manfro GG, Pan P, Rohde LA, Miguel EC, et al. Threat and deprivation are associated with distinct aspects of cognition, emotional processing, and psychopathology in children and adolescents. Dev Sci. 2023 Jan;26(1):e13267.
